# Innovative Monitoring Technologies to Enhance the Health, Independence, and Well-Being of Older Adults

**DOI:** 10.1093/geroni/igaf122.1765

**Published:** 2025-12-31

**Authors:** Walter Boot

## Abstract

Advancements in monitoring technologies offer new opportunities to enhance the health, independence, and well-being of older adults. This symposium presents innovative, non-invasive solutions for the early detection of functional decline, fall prevention, and support for aging in place. Dr. Megan Huisingh-Scheetz will discuss how hip accelerometry and machine learning models can detect and forecast frailty decline in older adults, enabling proactive interventions. Dr. Jeannette Mahoney will introduce CatchU... Before You Fall, a multisensory digital health tool designed to predict fall risk—particularly in individuals with preclinical Alzheimer’s disease—while promoting independence and facilitating provider-initiated falls counseling. Dr. Clara Berridge will explore the Let’s Talk Tech (LTT) decision aid, a web-based tool that helps individuals with memory loss and their caregivers make informed decisions about digital health technologies, highlighting its potential value to both family members and providers by supporting the sharing of technology preferences beyond the care dyad. Dr. Elinor Schoenfeld will present on the development and deployment of contactless sensors for Monitoring Vital Signs and Movement, advancing real-time health monitoring to support aging in place. Finally, Dr. Jane Chung will introduce a Wi-Fi Sensing-Based Deep Learning Solution that recognizes daily activities in older adults, offering insights into functional decline and cognitive health. By showcasing these cutting-edge approaches, this symposium highlights the transformative potential of monitoring technologies in aging research, emphasizing user-centered and scalable solutions to support older adults’ health and independence.

